# Automatic Evaluation of Voice Quality Using Text-Based Laryngograph Measurements and Prosodic Analysis

**DOI:** 10.1155/2015/316325

**Published:** 2015-06-02

**Authors:** Tino Haderlein, Cornelia Schwemmle, Michael Döllinger, Václav Matoušek, Martin Ptok, Elmar Nöth

**Affiliations:** ^1^Lehrstuhl für Mustererkennung, Friedrich-Alexander-Universität Erlangen-Nürnberg (FAU), Martensstraße 3, 91058 Erlangen, Germany; ^2^Klinik für Hals-, Nasen-, Ohrenheilkunde, Universitätsklinikum Magdeburg, Leipziger Straße 44, 39120 Magdeburg, Germany; ^3^Phoniatrische und Pädaudiologische Abteilung, Klinikum der Universität Erlangen-Nürnberg, Bohlenplatz 21, 91054 Erlangen, Germany; ^4^Department of Computer Science and Engineering, University of West Bohemia in Pilsen, Univerzitní 8, 306 14 Plzeň, Czech Republic; ^5^Klinik für Phoniatrie und Pädaudiologie, Medizinische Hochschule Hannover, Carl-Neuberg-Straße 1, 30625 Hannover, Germany

## Abstract

Due to low intra- and interrater reliability, perceptual voice evaluation should be supported by objective, automatic methods. In this study, text-based, computer-aided prosodic analysis and measurements of connected speech were combined in order to model perceptual evaluation of the German Roughness-Breathiness-Hoarseness (RBH) scheme. 58 connected speech samples (43 women and 15 men; 48.7 ± 17.8 years) containing the German version of the text “The North Wind and the Sun” were evaluated perceptually by 19 speech and voice therapy students according to the RBH scale. For the human-machine correlation, Support Vector Regression with measurements of the vocal fold cycle irregularities (CFx) and the closed phases of vocal fold vibration (CQx) of the Laryngograph and 33 features from a prosodic analysis module were used to model the listeners' ratings. The best human-machine results for roughness were obtained from a combination of six prosodic features and CFx (*r* = 0.71, *ρ* = 0.57). These correlations were approximately the same as the interrater agreement among human raters (*r* = 0.65, *ρ* = 0.61). CQx was one of the substantial features of the hoarseness model. For hoarseness and breathiness, the human-machine agreement was substantially lower. Nevertheless, the automatic analysis method can serve as the basis for a meaningful objective support for perceptual analysis.

## 1. Introduction

Voice is a perceptual phenomenon, and perceptual evaluation is therefore regarded as a gold standard for voice assessment [1, 2]. Hence, perception-based methods are the basis for the evaluation of voice pathologies in clinical routine, although they are too inconsistent among single raters to establish a standardized and unified classification [3, 4]. With this background of methodological shortcomings, simple rating criteria for perceptual evaluation have been established. Five of them have been combined to form the GRBAS scale [5] (grade, roughness, breathiness, asthenia, and strain). However, the choice of criteria has been criticized: asthenia (*A*) and breathiness (*B*) correlated very highly with each other in a study by Nawka et al., and the evaluation of the strain (*S*) criterion showed a much higher variation than the other criteria. For these reasons, the mentioned working group had developed a reduced version of GRBAS, the Roughness-Breathiness-Hoarseness (RBH) evaluation scheme [6]. It has become an established means for perceptual voice assessment in German-speaking countries.

Automatic, that is, computer-based, assessment may be helpful as an objective support for the subjective evaluation, since it omits the problem of intra- and interrater variation. Perception experiments are often applied to spontaneous speech, standard sentences, or standard texts. About automatic analysis, Maryn et al. reported that 18 out of 25 reviewed studies examined sustained vowels exclusively, four only speech, and three both vowels and speech [7]. For the analysis of speech, mostly one sentence of the English “rainbow passage” was used. Speech recordings have the advantage that they contain onsets, variations of *F*_0_, and pauses [8]. The impression of roughness, for instance, is influenced by the vowel onset fragments [9]. In general, hoarseness is more present and perceptible in long vowels, especially in open vowels, vowels in voiced context, vowels after glottal closure, or in strained vowels [10]. Hence, perceptual evaluation of a vowel and speech can only be adequately compared when the entire vowel with onset is evaluated [11, 12]. For automatic evaluation, some researchers recommend examining only the stable part of an isolated vowel [13], but following these recommendations means that a substantial portion of persons whose phonation is highly irregular cannot be evaluated at all. In particular, the rapid movements of the articulatory organs that are essential for the production of efficient speech require methods of analysis that go beyond the sole use of sustained vowels [14]. In order to diminish this problem, the Laryngograph has been designed to allow vocal fold closure to be monitored, most notably giving a basis for the measurement of aspects of vocal fold vibration which occur during voiced sounds [15].

In order to achieve a more global analysis of speech, the analysis of speech samples should be extended to methods that do not only evaluate voiced sounds. Also unvoiced sounds, words, the speaking rate, the duration and position of pauses within spoken phrases, the fundamental frequency and loudness, and their variations contribute to the complex phenomenon of speech. The analysis of these aspects has been subject of our working group in the field of automatic speech processing and understanding (identification of what was said and what it means) and also in automatic evaluation of voice and speech disorders (computer-based analysis of voice quality and speech properties, such as intelligibility). This analysis is achieved by a program package called the prosody module [16–18]. The goal of this work is to identify a computer-based equivalent for the subjective ratings of roughness, breathiness, and hoarseness from speech recordings, which are representative for communication by voice. This is achieved by means of the Laryngograph and prosodic analysis. Both systems of measurement are completely independent from each other.

Binary classification in the two classes “normal speech” and “pathologic speech” was not the goal of this study. Instead, the continuum of degrees of pathology and the continuum of human ratings were supposed to be modeled.

The questions addressed are the following.

How does the combination of prosodic analysis and Laryngograph measurements correspond with the perception-based RBH evaluation by “trained” listeners?

How do the results change when the Laryngograph measurements are left out or used as the only features for modeling the listeners' ratings?

## 2. Materials and Methods

### 2.1. Samples

**Figure 1:**
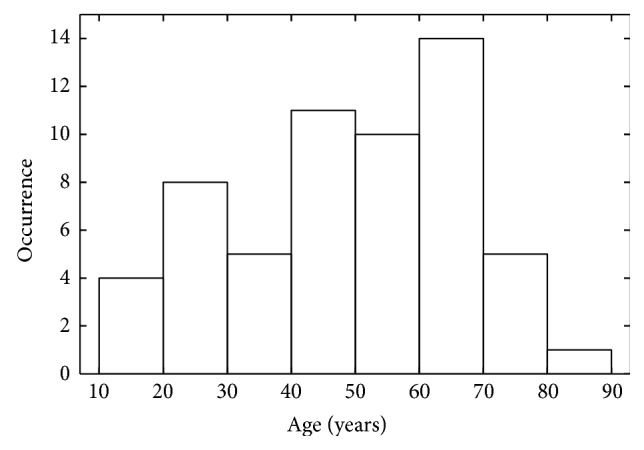
Age distribution of the speaker group (*n* = 58).

58 speech samples (43 samples of female and 15 samples of male voices) were used in this study. The age of the persons was between 12.2 and 81.9 years and the average age was 48.7 years with a standard deviation of 17.8 years. The age distribution is shown in Figure 1. The speech samples were recorded at the Medical University Hannover, Department of Phoniatrics and Pedaudiology, within an interval of three months. Only the set of recordings that was acquired during the first visit at the clinics was used of each person. The collection of samples was supposed to be representative, so no further selection was made. For this reason, the database contained deviated voices and also “normal” voices (Table 1). The most frequent pathology was dysphagia (*n* = 16). The subjects were examined by experienced laryngologists and phoniatricians following the standard protocol of the European Laryngological Society [19].

The speech samples contained connected speech, namely, the standard text “Der Nordwind und die Sonne” (“The North Wind and the Sun”) [20] which is frequently used in medical speech evaluation in German-speaking countries. The version used for this study consisted of 109 words. The recordings were made with components of the Laryngograph system [21]. The headset of the system was placed at a distance of 10 cm in front of the reader's mouth. The speech data were recorded with a sampling frequency of 44.1 kHz and a 16 bit amplitude resolution. For automatic speech analysis, the data were resampled with a 16 kHz sampling frequency. In order to obtain the other Laryngograph measurements, two electrodes were placed superficially on either side of the neck of the subject at the level of the larynx, and a constant amplitude high-frequency voltage (3 MHz) was applied. This setup was chosen in order to ensure conditions which are usual in clinical applications.

The study has respected the principles of the World Medical Association (WMA) Declaration of Helsinki on ethical principles for medical research involving human subjects. All patients had given written consent to the anonymized use of their data for research purposes before the recordings.

### 2.2. Perceptual Evaluation

The perceptual evaluation of the text recordings according to clinical standards was done by 19 speech and voice therapy students (3rd year female students, study course on speech therapy at the Fresenius University of Applied Sciences, Idstein, Germany) using the RBH scale [6]. The students had learned about the RBH scheme from the beginning of their education. In the third year, they have sufficient theoretical and practical knowledge about voice evaluation, the ability to interpret larynx-related diagnoses, and practical experience, since they have also undergone practical training including therapy lessons by themselves under supervision.

Before the listening task, detailed instruction was given to the students by the study tutors. During the task, no further information was given, however. The raters listened to each speech sample once. This was sufficient since the duration of one recoding was 46 seconds on the average. Between two samples, there was a pause to note down the results. The students were not allowed to discuss their impression with the other raters.

For one speech sample, each of the RBH criteria, that is, roughness, breathiness, and hoarseness, can be evaluated on a 4-point scale where “0” means “absent” and “3” means “high degree.” Originally it was believed that hoarseness is distinct of the other two categories, roughness and breathiness [22]. The RBH instead assumes that hoarseness is a superclass of them [23]. In order to capture the fact that hoarseness is the superclass, the *H* rating value must usually be at least as high as *R* and *B*. For this study, however, this latter rule was not applied, and the students were told to evaluate hoarseness on the 4-point scale just by their impression of the replayed speech. This procedure has already been performed in several other studies in Germany [24–26].

### 2.3. Laryngograph Measurements

The Laryngograph measures the time and degree of contact between the vocal folds by the application of two electrodes which are placed on the neck. The electroglottogram serves as the basis for the computation of several measures. Two of them have been used in this study and will be explained below. Although the voiced excitation of the vocal tract is a complex activity, it has two main time-dependent characteristics. The first one is derived from the duration of excitation of the vocal tract, when the closure of the vocal folds produces its main acoustic signal; the second one relates to the period during which the vocal folds are effectively closed [21]. The fundamental frequency (*F*_0_) is usually estimated from short-time windows and based on average values from several vocal fold cycles, which may also be fragmented at the boundaries of the analysis window. A period-synchronous analysis is more exact since it takes into account only full cycles and can also consider period-to-period variations that are often of perceptual importance. These variations of the period frequency values Fx are denoted as CFx in the Laryngograph software. Another measuring factor, which provides information about perceived voice quality, is the changes CQx of the contact phase Qx. The latter is directly related to the ratio of the closed phase of vocal fold vibration to the total period of time between two successive epochs of excitation [21]. In this study, CFx and CQx were used in combination with prosodic features to describe voice quality. Both values are given in percent.

### 2.4. Prosodic Features

The computation of the prosodic features is independent from the Laryngograph. A speech recognition system [27] detects the spoken words and their positions in the speech recordings. Then the prosodic analysis module [16] computes a vector of prosodic features for each word. There are three basic groups of features. Duration features represent word and pause durations. Energy features contain information about maximum and minimum energy, their respective positions in the word, the energy regression coefficient, and the mean square error. Similarly, the *F*_0_ features, based on the detected fundamental frequency, comprise information about the extreme *F*_0_ values and their positions, voice onset and offset with their positions, and also the regression coefficient and the mean square error of the *F*_0_ trajectory. Duration, energy, and *F*_0_ values are stored as absolute and as normalized values. The basic features are computed in different contexts, that is, in intervals containing a single word or pause only or a word-pause-word interval. In this way, 33 features were computed for each word (see Table 2) [17, 28, 29].

Besides the 33 local features per word, 15 “global” features were computed for intervals of 15 words length each. They were derived from jitter (fluctuations of *F*_0_), shimmer (fluctuations of intensity), and the number of detected voiced and unvoiced sections in the speech signal [28]. They covered the means and standard deviations of jitter and shimmer, the number, length, and maximum length of voiced and unvoiced sections, the ratio of the numbers of voiced and unvoiced sections, the ratio of the length of the voiced sections to the length of the signal, and the same for unvoiced sections. The last feature was the standard deviation of *F*_0_.

The listeners gave ratings for the entire text. In order to receive also one single value for each feature that could be compared to the human ratings, the average of each prosodic feature over the entire recording served as final feature value.

### 2.5. Support Vector Regression

A Support Vector Machine (SVM) performs a binary classification based on a hyperplane separation between two class areas in a multi-dimensional feature space. SVMs can also be used for Support Vector Regression (SVR) [30]. The general idea of regression is to use the element vectors of the training set to approximate a function which tries to predict the target value of a given vector of the test set. In this study, the sequential minimal optimization algorithm (SMO) [30] of the Weka toolbox [31] was applied for this purpose. The automatically computed prosodic features and the CFx and CQx values served as the training set for the regression, and the test set consisted of the perceptually assessed RBH scores. For each of *R*, *B*, and *H*, one separate regression was computed.

In order to find the best subset of the computed features to model the subjective ratings, a correlation-based feature selection method ([32], pp. 59–61) was applied in a 10-fold cross-validation manner. The features with the highest ranks were then used as the input for the SVR.

### 2.6. Human-Machine Correlation

Statistical analysis was performed using Weka and in-house programs. The interrater reliability for the entire rater group was measured using Krippendorff's *α* [33]. Many studies use Cronbach's *α*, but this measure eliminates the influences of different tendencies in rating since the mean values are neglected. In order to examine human-machine correlation, the automatic measurement for each rating criterion of each recording was compared to the average value of the 19 raters' evaluation. The correlations between different measurements and rating criteria were computed using Pearson's correlation coefficient *r* and Spearman's rank-order correlation coefficient *ρ*. Other measures, like Cohen's *κ* or Krippendorff's *α*, were not used for this purpose due to the different domains of human and machine evaluation. This means, for instance, that continuous intervals of the prosodic features or the Laryngograph values would have to be mapped to the discrete values {0,1, 2,3} of the RBH components, which is another possible source of error [34].

## 3. Results

### 3.1. Perceptual Data

**Figure 2:**
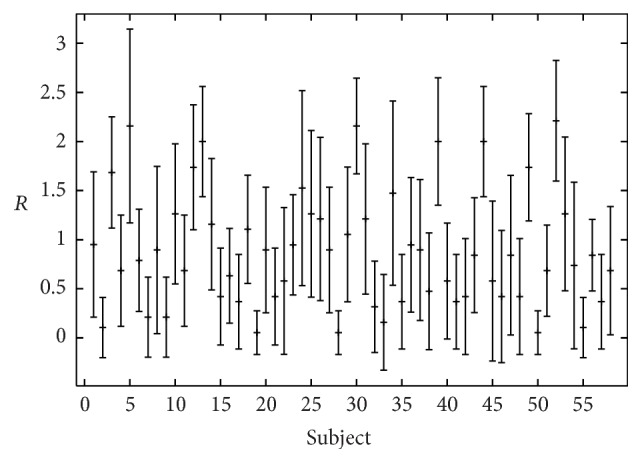
Perceptual roughness (*R*) evaluation by 19 listeners (mean value and standard deviation).

**Figure 3:**
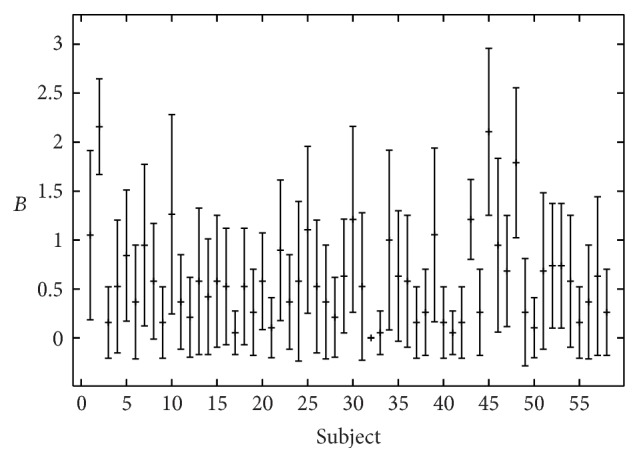
Perceptual breathiness (*B*) evaluation by 19 listeners (mean value and standard deviation).

**Figure 4:**
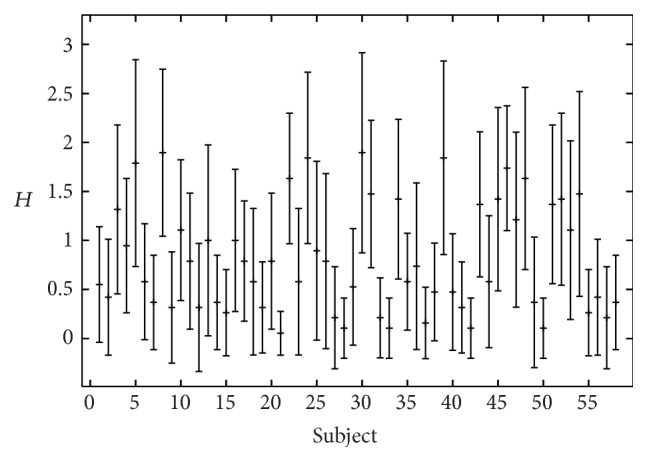
Perceptual hoarseness (*H*) evaluation by 19 listeners (mean value and standard deviation).

The average values for the perceptual rating criteria are given in Table 3. The data showed a broad range of persons with minimal values of *R*_min_ = 0.05, *B*_min_ = 0.00, and *H*_min_ = 0.05, respectively, to maximum values for *R*, *B*, and *H* around 2. A large variety in the evaluation results was observed within the rater group as well (Figures 2–4). The interrater values for the 19 listeners were *α* = 0.45 for roughness, *α* = 0.33 for breathiness, and *α* = 0.36 for hoarseness (Table 3). Correlations between the rating criteria are given in Table 4. The criteria roughness and breathiness correlate only moderately with each other. The strongest correlation is between breathiness and hoarseness (*r* = 0.53, *ρ* = 0.67).

### 3.2. Human-Machine Correlation

**Figure 5:**
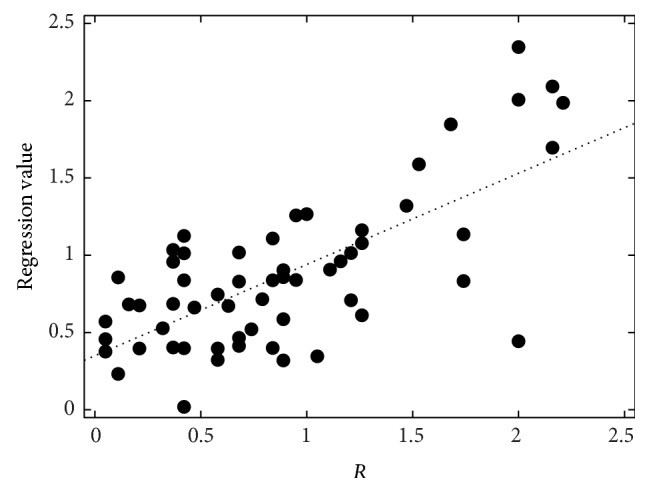
Perceptual roughness (*R*) evaluation by 19 listeners, the SVR regression values (*R*_best,I_), and their best-fit line.

**Figure 6:**
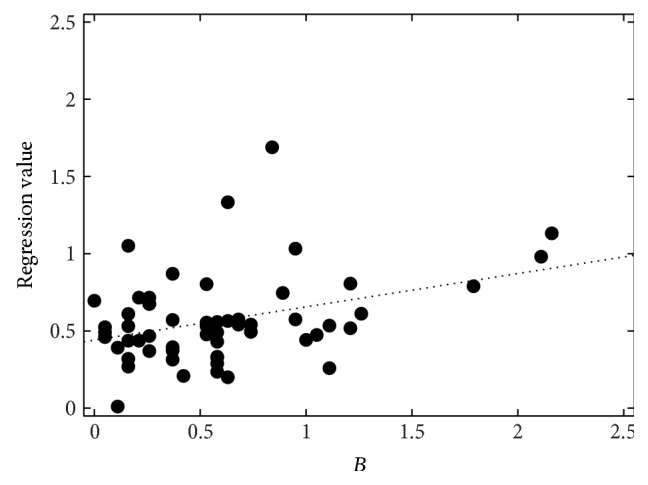
Perceptual breathiness (*B*) evaluation by 19 listeners, the SVR regression values (*B*_best_), and their best-fit line.

**Figure 7:**
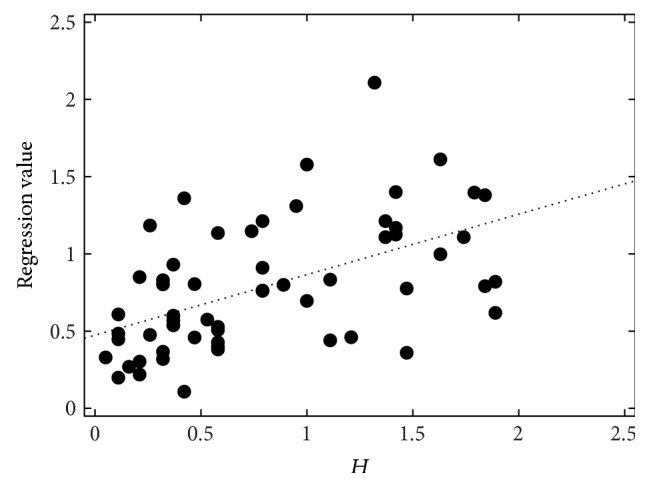
Perceptual hoarseness (*H*) evaluation by 19 listeners, the SVR regression values (*H*_best,I_), and their best-fit line.

The correlations between the perceptual evaluation and the automatic measurements after the SVR are given in Table 5. The best set for roughness (*R*_best,I_) achieves *r* = 0.71 (*ρ* = 0.57). It contains the duration of a word-pause-word interval (DurNormWPW), the mean and minimum *F*_0_ within a word (F0MeanW, F0MinW), mean jitter and shimmer averaged on 15-word sections (MeanJitter, MeanShimmer), the number of sections detected as voiced (#+Voiced), and CFx. Without CFx, only *r* = 0.66 (*ρ* = 0.49) is reached (set *R*_best,I_ w/o CFx). The duration feature can also be left out without changing the correlations significantly (sets *R*_best,II_ and *R*_best,II_ w/o CFx). The same feature is in the best set for breathiness modeling (*B*_best_), which, however, was far less successful in modeling the reference with *r* = 0.36 (*ρ* = 0.27). Still, this correlation is highly significant. Neither CFx nor CQx are included in the breathiness model. For hoarseness, there are four different results, denoted *H*_best,I_ to *H*_best,IV_. The best correlation is *r* = 0.53 (*ρ* = 0.54) for a combination of word duration (DurNormW), the voice offset position within single words (F0OffPosW), the normalized energy within words (EnNormW), the “global” number of voiced sections in the recording (#+Voiced), and the ratio between the numbers of voiced and unvoiced sections (RelNum+/−Voiced). CQx is also essential for the best feature set for hoarseness. Without CQx, the set *H*_best,I_ reaches only human-machine correlations of about 0.35; with CFx instead of CQx, the highest values are below 0.5. Figures 5–7 show the perceptual evaluations, that is, the average of the 19 raters, and the regression values of the SVR for the best feature sets.

**Figure 8:**
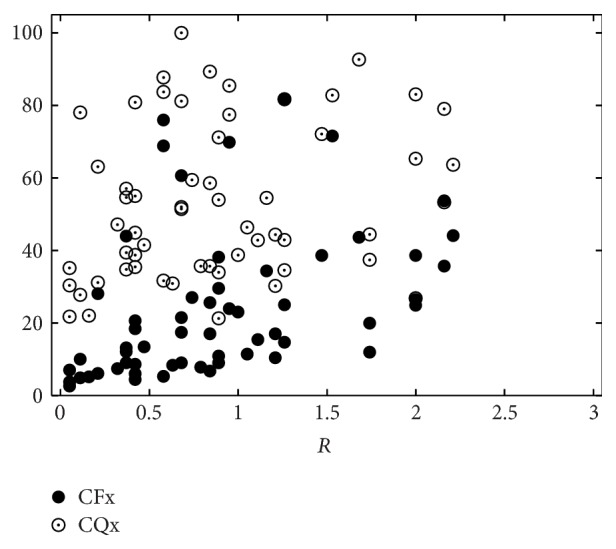
Perceptual roughness (*R*) evaluation by 19 listeners versus CFx and CQx, respectively.

**Figure 9:**
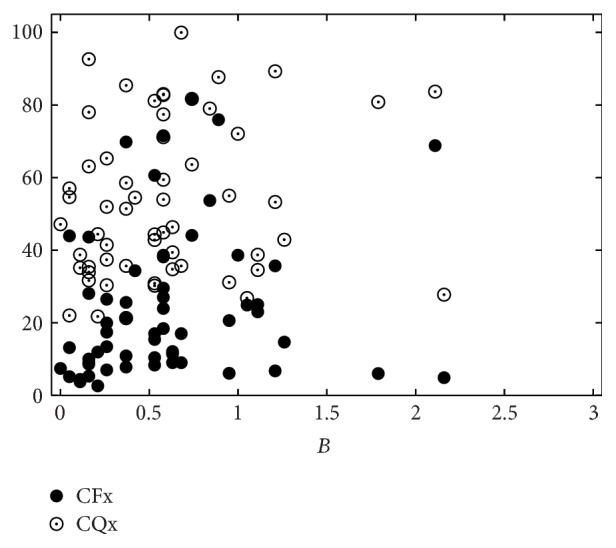
Perceptual breathiness (*B*) evaluation by 19 listeners versus CFx and CQx, respectively.

**Figure 10:**
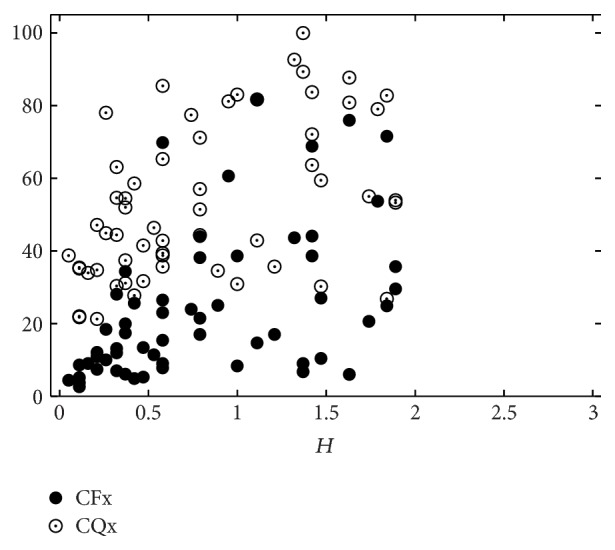
Perceptual hoarseness (*H*) evaluation by 19 listeners versus CFx and CQx, respectively.

Table 6 shows the human-machine correlations for combinations of CFx and CQx only. These two measures can model the perceptual impression of hoarseness moderately (*r* = 0.44, *ρ* = 0.48), while they are only weakly correlated with roughness and breathiness. The distribution of these measurements is shown in Figures 8–10.

## 4. Discussion

The Laryngograph is an established means of voice evaluation [14, 35]. The main purpose of this study was to determine the correlation between the German RBH evaluation scheme and a combination of text-based prosodic features and measurements from the Laryngograph. The best combination of features yielded a human-machine correlation for roughness of *r* = 0.71 (*ρ* = 0.57). The interrater correlation for one rater against the average of all others was *r* = 0.65 (*ρ* = 0.61). Hence, the automatic analysis can evaluate roughness as reliable as an “average” rater from the group of the 19 speech and voice therapy students. For hoarseness, the automatic method reached almost the same correlation with the reference as the listeners among themselves. Only the breathiness rating could not be modeled satisfactorily. Additionally, dropping one of the feature sets from the automatic evaluation leads to significantly worse results.

For the modeling of roughness, the duration of a word-pause-word interval (DurNormWPW) may contribute to the most successful set of features because the anatomic alterations, which are the reason for the deviated voice, may also cause a greater speaking effort. This effect has been shown for substitute voices of laryngectomized persons [17], and it might also be valid for the data in this study. The contribution of DurNormWPW to the regression sum is, however, very small.

The impact of the values F0MinW and F0MeanW can be explained by the properties of the *F*_0_ detection algorithm, which does a voiced-unvoiced decision first. On all of the 16 ms speech frames that were classified as voiced, the program performed *F*_0_ detection. The algorithm by Bagshaw et al. [36] that was used for the task is very robust against distortions. However, noisy speech may result in octave errors, that is, instead of the real fundamental frequency the double, triple, or half of the actual value is found. More “noisy” speech influences the *F*_0_ trajectory and thus the correlation with the subjective results [18].

A similar case is the relevance of text-based jitter and shimmer for the model of the roughness evaluations. Both are well-known detectors for voice problems, and the number of segments in the recording which were detected as voiced corresponds with these findings. If a voice is very irregular, then the number of segments detected as voiced by the prosody module will be very low. A difficulty for the comparison of these results with other studies, however, is that the terms “jitter” and “shimmer” disguise a plethora of different algorithms, across many different software vendors and research groups [37]. Many studies give no algorithm details. Additionally, irregularity measures from sustained, isolated vowels and running speech cannot be directly compared due to coarticulatory effects and differences in voice onset and offset.

In this study, also the CFx value appeared to be essential for the good human-machine correlation for roughness. When it was missing, the correlation dropped down to *r* = 0.66 (*ρ* = 0.49). CFx is also related to variations of *F*_0_, but it is period-synchronous instead of being based on fixed-length windows. That is on the one hand an advantage against the traditional computation of jitter. On the other hand, the low correlation between CFx and jitter values (Table 7) indicates that both are containing important but independent information.

Breathiness can be modeled only weakly by the available features. While the human-human correlation was *r* = 0.58 (*ρ* = 0.50), the maximum for the automatic analysis was *r* = 0.36 (*ρ* = 0.27). Here, the duration of a word-pause-word interval contributes very strongly. The reason may be that the continuous leaking of air at the glottis leads to longer or more frequent pauses.

The contribution of the *F*_0_ value at voice onset (F0OnsetW) may be based upon octave errors by the *F*_0_ detection algorithm again. So far, it is not clear why only the beginning of voiced sections causes a noticeable effect. There may be a connection to changes in the airstream between the beginning and end of words or phrases. It may have its reason in the high speaking effort in the dysphonic voice which leads to more irregularities, especially in these positions, but this has to be confirmed by more detailed experiments on larger and homogeneous databases.

The influence of the normalized energy in the breathiness model was only relevant when it was measured within one word (EnNormW) and not in a word-pause-word interval. Hence, breathing noise in pauses does not contribute to the result, although the duration of the pauses may be important, as pointed out above. The sign of the weighting factor (−0.247) is negative, so the breathier the voice is, the weaker it is and the higher the human *B* evaluation is.

Jitter is also an important factor for the evaluation of breathiness; however, not all authors of other studies agree [38, 39]. Shimmer shows only a very low contribution, but the standard deviation of shimmer within longer text passages, that is, the fluctuations of the fluctuations of energy, seems to be characteristic for breathiness.

Neither CFx nor CQx were in the optimal set for breathiness evaluation.

For hoarseness, many features were in the best subsets that were also relevant for roughness and breathiness. This supports the assumption of Nawka et al. that hoarseness is a superclass of the other two criteria [6], although the students did not evaluate the data with this rule in mind explicitly. The feature set modeling the raters' decisions best reached a correlation of *r* = 0.53 (*ρ* = 0.54) to that reference; the interrater correlation was *r* = 0.59 (*ρ* = 0.57). Like for breathiness, the duration is important, but only on single words, not on word-pause-word intervals. Replacing the feature with the latter variant yields much worse correlations (Table 5, column *H*_best,III_), as did using the word-based feature for modeling roughness.

The normalized energy within words (EnNormW) is, like for breathiness, another important feature. Replacing it with the word-pause-word variant (EnNormWPW) was not successful (Table 5, columns *H*_best,II_ and *H*_best,IV_).

The average of jitter contributes to the hoarseness model even more than to the two other categories.

The position of the voice offset within a word (F0OffPosW), which did not occur in the roughness and breathiness modeling, is a nonnegligible factor for hoarseness evaluation. This has already been detected in a previous study with chronically hoarse persons who were evaluated by five voice experts [18]. The reason is very probably the *F*_0_ detection algorithm and its decisions regarding voiced and unvoiced sections again.

Shimmer was not relevant for hoarseness at all in the results, although it showed contributions to the regression sum of roughness and breathiness. This supports, in contrast to Nawka's assumption, the hypothesis that hoarseness may be more than just the superclass of the other categories.

As with roughness, the number of sections that are classified as voiced (#+Voiced) is important for hoarseness evaluation. Additionally, the ratio of the numbers of voiced and voiceless sections (RelNum+/−Voiced) supports the results.

The high correlation of perceptual *B* and *H* evaluations shows that for the evaluation of overall hoarseness the raters were closer to the breathiness rating than to the roughness rating. This is in contrast to another study of our group, where roughness and hoarseness had a higher correlation (*ρ* = 0.79) [34]. For that study, however, the restriction *H* ≥ max⁡(*R*, *B*) was applied, and only five speech therapists with several years of experience in voice evaluation had rated the data. In this new study, there was also a large variety of ratings among the 19 listeners. Therapists with many years of practical experience may show less disagreement [40], but according to the fact that the raters of our study had undergone almost three years of practical education before, we believe that they already developed a rather stable personal model of voice evaluation. The influence of these factors on our particular data has to be examined in future work.

The automatic modeling of the hoarseness and especially the breathiness ratings was not as successful as for roughness. The set of available measures and prosodic features was not sufficient to depict the various ratings of the large rater group satisfyingly so far. Nevertheless, the method presented here may be the basis for a meaningful objective support and an addition to perceptual analysis in clinical practice. Another important advantage of the presented method is that it does not just classify voices into one of the two categories “normal” and “pathologic.” For quantification of a communication disorder in clinical use, this is not sufficient. Instead, the experiments provided regression formulae which can be used to translate the obtained measures onto the whole range of perceptual ratings.

A complete match of subjective and automatic evaluation was not expected. On the one hand, disagreement on which acoustic properties or measures represent which perceptual impression may still be present; on the other hand the automatic assessment can only be based on a stimulus which for perceptual evaluation is further processed within the listener. Hence, the sources of information for both methods are different. The process of perception may evaluate more or different information than the automatic methods. Additionally, there is also some possible improvement for the technical methods which is part of future work. As an example, the speech recognition module, which is supposed to provide the word hypotheses graph for the computation of the prosodic features, can be improved by adaptive methods to enhance the phoneme models for distorted speech [41]. For these reasons, we regard this study as a pilot study. Furthermore, the automatic evaluation is not supposed to be a full replacement for the subjective assessment, but an additional source of information which yields reproducible results.

## 5. Conclusions

Combined prosodic and Laryngograph-based analysis corresponds as well with the average perception-based roughness evaluation as a group of professional raters themselves on a clinical representative group of patients with a broad distribution of voice pathology. It can serve as an additional source of knowledge or an objective guideline in the clinics where perceptual evaluations are usually performed by a single person only.

## Figures and Tables

**Table 1:** Diagnoses within the speaker group (*n* = 58).

Edema	
Reinke's edema (bilateral)	3
Edge edema	1

Pareses	
Vocal fold paresis (right)	8
Vocal fold paresis (left)	3
Vocal fold paresis (bilateral)	2

Benign tumors, pseudotumors	
Hyperplasia vocal fold (right)	1
Vocal fold polyp (right)	4
Vocal fold polyp (left)	1
Vocal fold cyst (right)	1
Vocal fold nodules	3
Vocal fold granuloma	1
Larynx papillomatosis	1

Inflammations	
Laryngitis	3

Central movement disorders	
Spasmodic dysphonia	3
Balbuties	1
Other central disorders	1

Functional dysphonia	
Psychogenic dysphonia	1
Dysphagia	16
Normal laryngeal findings	4

**Table 2:** Prosodic features and their intervals of computation; 33 prosodic features are based upon duration (“Dur”), energy (“En”), and fundamental frequency (“F0”) measures. The context size denotes the interval of words on which the features are computed; W: computed on current word, WPW: computed in the interval that contains the second and first word before the current word, and the pause between them.

Features	Context size	
	WPW	W
Pause: before, Fill-before, after, Fill-after		•
En: RegCoeff, MseReg, Abs, Norm, Mean	•	•
En: Max, MaxPos		•
Dur: Abs, Norm	•	•
F0: RegCoeff, MseReg	•	•
F0: Mean, Max, MaxPos, Min, MinPos, Off, OffPos, On, OnPos		•

The features are abbreviated as follows.

Length of pauses “Pause”: length of silent pause before (before) and after (after), and filled pause before (Fill-before) and after (Fill-after) the respective word in context.

Energy features “En”: regression coefficient (RegCoeff) and mean square error (MseReg) of the energy curve with respect to the regression curve; mean (Mean) and maximum energy (Max) with its position on the time axis (MaxPos); absolute (Abs) and normalized (Norm) energy values.

Duration features “Dur”: absolute (Abs) and normalized (Norm) duration.

*F*_0_ features “F0”: regression coefficient (RegCoeff) and the mean square error (MseReg) of the *F*_0_ curve with respect to its regression curve; mean (Mean), maximum (Max), minimum (Min), voice onset (On), and offset (Off) values as well as the position of Max (MaxPos), Min (MinPos), On (OnPos), and Off (OffPos) on the time axis; all *F*_0_ values are normalized.

**Table 3:** Perceptual evaluation results (average, standard deviation, and minimal and maximal values) and interrater agreement expressed as Krippendorff's *α* and the correlation coefficients *r* and *ρ* (*n* = 58).

	Average	Standard dev.	Min	Max	*α*	*r*	*ρ*
*R *	0.88	0.51	0.05	2.21	0.45	0.65	0.61
*B *	0.59	0.47	0.00	2.16	0.33	0.58	0.50
*H *	0.81	0.56	0.05	1.89	0.36	0.59	0.57

**Table 4:** Correlation *r* (*ρ*) between the perceptual ratings (*n* = 58).

	*B *	*H *
*R *	0.13 (0.33)	0.50 (0.53)^*∗*^
*B *		0.53 (0.67)^*∗*^

*∗* = correlation is significant on the 0.01 level.

**Table 5:** Best feature sets for human-machine correlation and their weights in the regression formulae.

Feature	Context	R_best,I_	R_best,I_ w/o CFx	R_best,II_	R_best,II_ w/o CFx	B_best_	H_best,I_	H_best,II_	H_best,III_	H_best,IV_
DurNorm	WPW	−0.057	−0.046			0.377		0.499	0.378	
DurNorm	W						0.513			0.402
F0Min	W	−0.446	−0.458	−0.452	−0.389					
F0Mean	W	−0.195	−0.226	−0.191	−0.172					
F0Onset	W					0.173				
F0OffPos	W						0.322	0.120	0.185	0.236
EnNorm	WPW							−0.151		0.343
EnNorm	W					−0.247	−0.315		0.155	
MeanJitter	15 W	0.118	0.186	0.113	0.249	0.239	0.366	0.368	0.320	0.208
MeanShimmer	15 W	0.144	0.138	0.145	0.114	−0.031				
StandDevShimmer	15 W					−0.163				
#+Voiced	15 W	0.321	0.347	0.334	0.324		0.094	−0.133	−0.117	0.122
RelNum+/−Voiced	15 W						−0.164	0.218	0.082	−0.144
CFx		0.210		0.206						
CQx							0.643	0.495	−0.242	0.506

*r*		0.71	0.66	0.71	0.67	0.36	0.53	0.47	0.45	0.49
*ρ*		0.57	0.49	0.58	0.49	0.27	0.54	0.46	0.45	0.55
Significance level		<0.001	<0.001	<0.001	<0.001	0.003	<0.001	<0.001	<0.001	<0.001

Contexts: W: word, WPW: word-pause-word, 15 W: 15 words (“global” feature). The correlations of the respective set to the human reference are given by *r* (Pearson) and *ρ* (Spearman).

**Table 6:** Weighting factors in the regression sums when the *RBH* rating is modeled by CFx and CQx only and the human-machine correlation (*r*, *ρ*).

Feature	*R *	*B *	*H *
CFx	0.303	0.091	0.340
CQx	0.033	0.117	0.490

*r*	0.31	−0.10	0.44
*ρ*	0.43	−0.05	0.48
Significance level	0.009	0.228	<0.001

**Table 7:** Correlations of prosodic and Laryngograph measures, which were in the best models for the human rating, with each other.

Feature		DurNorm	DurNorm	F0Min	F0Mean	F0Onset	F0OffPos	EnNorm	EnNorm	MeanJitter	MeanShimmer	StandDevShimmer	#+Voiced	RelNum+/−Voiced	CFx	CQx
	Context	WPW	W	W	W	W	W	WPW	W	15 W	15 W	15 W	15 W	15 W		
DurNorm	WPW		0.02	−0.17	−0.04	0.01	−0.23	**0.93**	0.03	0.07	−0.03	−0.12	0.01	−0.05	0.20	0.04
DurNorm	W	0.10		**−0.30**	−0.24	−0.19	**−0.30**	0.01	**0.78**	0.22	0.00	−0.03	0.08	0.13	0.13	0.05
F0Min	W	0.02	**−0.30**		**0.53**	**0.56**	**0.34**	−0.19	−0.11	**−0.54**	**−0.31**	−0.14	**−0.70**	**−0.58**	**−0.26**	−0.11
F0Mean	W	−0.09	**−0.31**	**0.62**		**0.68**	**0.39**	−0.02	−0.09	−0.07	**−0.32**	0.02	−0.17	−0.13	−0.03	0.12
F0Onset	W	0.00	−0.23	**0.62**	**0.62**		**0.29**	0.05	−0.06	−0.01	−0.12	0.07	−0.10	−0.10	0.02	0.06
F0OffPos	W	−0.13	**−0.32**	**0.33**	0.20	**0.27**		−0.18	−0.23	−0.04	**−0.26**	0.08	**−0.32**	**−0.32**	−0.01	0.01
EnNorm	WPW	**0.92**	0.07	0.02	−0.06	0.07	−0.11		0.06	0.14	0.02	−0.10	0.08	0.00	0.14	0.00
EnNorm	W	0.19	**0.68**	−0.11	−0.13	−0.03	−0.22	0.24		0.15	−0.14	−0.11	−0.02	−0.02	0.07	0.02
MeanJitter	15 W	−0.11	0.18	**−0.30**	−0.11	0.00	0.08	−0.04	0.03		**0.40**	**0.38**	**0.62**	**0.57**	**0.35**	0.23
MeanShimmer	15 W	−0.18	−0.02	**−0.27**	**−0.36**	−0.13	−0.23	−0.08	−0.20	0.21		**0.75**	**0.43**	**0.40**	0.15	−0.04
StandDevShimmer	15 W	**−0.28**	−0.10	−0.10	−0.03	−0.02	−0.03	−0.21	−0.16	0.17	**0.75**		**0.34**	**0.37**	0.15	0.04
#+Voiced	15 W	−0.13	0.13	**−0.63**	**−0.29**	−0.21	**−0.35**	−0.06	0.00	**0.51**	**0.34**	**0.31**		**0.89**	**0.30**	0.15
RelNum+/−Voiced	15 W	−0.14	0.16	**−0.56**	−0.24	−0.17	**−0.30**	−0.09	−0.01	**0.51**	**0.31**	**0.31**	**0.93**		0.20	0.07
CFx		0.08	0.24	**−0.35**	−0.07	−0.02	−0.18	0.05	0.10	**0.40**	0.12	0.00	**0.45**	**0.37**		**0.65**
CQx		−0.07	0.02	−0.04	0.18	0.07	−0.01	−0.12	−0.04	0.15	−0.03	−0.04	0.11	0.08	**0.64**	

Upper right triangle: Pearson's *r*; lower left triangle: Spearman's *ρ*.

Contexts: W: word, WPW: word-pause-word, 15 W: 15 words (“global” feature).

All *r* and *ρ* correlations with an absolute value of larger than 0.25 (0.33) are significant on the 0.05 (0.01) level.
